# The mechanical ventilator of the future: a breath of hope for the viral pandemics to come

**DOI:** 10.11604/pamj.2022.41.321.29726

**Published:** 2022-04-20

**Authors:** Luiz Alberto Cerqueira Batista Filho

**Affiliations:** 1 Department of Research, Imed Group Brasil, Avenida Angelica, Bela Vista, São Paulo/SP, 01228-200, Brazil

**Keywords:** Artificial intelligence, machine learning, mechanical ventilation, critical care, COVID-19

## Abstract

Respiratory care for the critically ill is a complex and difficult duty to accomplish. By replicating human knowledge with automated algorithms, artificial intelligence could provide solutions to facilitate this multidisciplinary task in developing countries, especially during humanitarian crisis, as the COVID-19 pandemic. This article provides an overview on the subject, from the emergent nations perspective.

## Commentary

“Ventilators are not the answer in Africa”. This is the title of an editorial [1] written in 2020 by a physician from Zimbabwe, emphasizing that Africa has been struggling with the lack of oxygen infrastructure, electricity and many other resources, during the fight against COVID-19. Many professionals are not proficient in caring for the critically ill, contributing to the high mortality amongst those who need mechanical ventilation, especially in developing countries [2]. Beyond any doubt, intelligent devices could be of great help in facilitating the respiratory care of these patients, by helping physicians to make better decisions. In the last decades, many countries have been setting up the grounds for machine learning in healthcare, recording information about every aspect of patients in big databases, colloquially known as *big data*.

By using advanced statistical techniques, such as data mining, big data analysis identifies patterns within the data, offering substratum for *machine learning* (ML). Machine learning can be supervised, unsupervised or mixed [3]. During supervised learning, the *automated algorithm* knows if the patient has the studied disease or not, and will look for patterns within the database in order to determine which are the variables that will predict the development of this specific health condition. The user *supervises* ML by offering the program solved problems. With unsupervised learning, the outcome is not given to the program, and it will look for patterns within the data, finding risk groups for the studied condition. In this case, the machine is not instructed about the type of answer to produce. With semi-supervised or mixed ML, the algorithm has access to a mix of solved and unlabeled data, and it tries to learn from uncategorized data, increasing its performance to diagnose or prognosticate.

Noticeably, detecting and treating patient-ventilator asynchronies (PVA) is one of the most challenging tasks for doctors in critical patients. Patient-ventilator asynchronies are defined as mismatch between the patient´s inherent inspiratory and expiratory times. The most common approach to correcting PVAs is to increase sedation, but there is evidence that this strategy is also associated with increased length of stay, delirium and mortality [4]. Assessment of PVAs can be done with various techniques, which include analysis of flow-time, pressure-time, and integrated volume-waveforms from ventilator screens. More recently, the use of automated algorithms has been in the center of discussions. In 2018, investigators made an experiment [5], where an algorithm was taught how to detect PVAs by being offered the results of the analysis of 1378 breath cycles waveforms, made by five experienced human experts. This supervised ML investigation showed that the proposed framework accurately detected absence of cycling asynchrony with a sensitivity (specificity) of 89% (99%), 94% (98%) and 97% (93%) for delayed termination, premature termination, and no cycling asynchrony, respectively. This study establishes feasibility of using machine learning framework to provide waveform analysis equivalent to a human expert.

Evaluation of lung mechanics is essential to ensure fully protective ventilation. Treating acute respiratory distress syndrome involves ventilating with volumes ≤6ml/kg of predicted body weight, plateau pressure ≤ 30 cm/H_2_O and driving pressure ≤ 15 cm/H_2_O, aiming to avoid ventilator-induced lung injuries. Each patient has a state of disease, and ventilation should be optimized accordingly. Calculating respiratory resistance (R) and compliance (C) is crucial, and many doctors are not familiar with the maneuvers to obtain these variables, or with the basic concepts of lung mechanics. A machine learning algorithm was developed to track flow rate and airway pressure in order to estimate R and C continuously and in real-time, using a test lung [6]. It showed a 99.4% accuracy using a linear regression model, demonstrating good potential.

By mechanically ventilating ten pigs, investigators used an artificial neuron network (ANN) to calculate the total pressure exerted on the alveolar walls (PEEP_tot_) [7]. PEEP_tot_ is calculated by the sum of the positive airway pressure at the end of expiration (PEEP_app_) and intrinsic PEEP (PEEPi), a hidden component. To evaluate PEEP_tot_, is necessary to perform an end-expiratory maneuver (EEHM), and the investigators hypothesized that the ANN could estimate the variable from flow and pressure tracings during ongoing mechanical ventilation. Different respiratory patterns (R_pat_) were obtained, and an EEHM was performed in the last breath of each R_pat_, as the value was used as a reference. Acute lung injury was induced with the use of intravenous injection of oleic acid, and new R_pat_ were obtained. The ANN had to extract the PEEP_tot_ from the tracings, without EEHM, and it showed good agreement with the reference PEEP_tot_, appointing to a new path of calculating PEEP_i_. One of the problems related to mechanical ventilation is the patients´ loss of the ability to eliminate secretions from the airways and the occurrence of ventilator associated pneumonia, with increased mortality. Artificial secretion clearance is absolutely fundamental in the Intensive Care Unit (ICU), and sputum sounds could be useful as biological signals generated by the vibration of sputum in respiration. A chinese study [8], carried out in 2018, collected and recorded respiratory sound signals in intubated patients. The sound samples were classified as sputum deposition and non-sputum deposition signals by specialists and processed for noise cleansing. Following pretreatment for noise removal, the sounds were decomposed by wavelet transform, obtaining features of the signals, represented by wavelet coefficient distributions. These patterns created a dataset of 595 samples that was used to train ANNs into recognizing sputum deposition sounds using a Back Propagation algorithm. Back propagation neural networks uses training samples to establish weights and thresholds to adjust and minimize the mean square error of the network output value. In this paper, the output was sputum or non-output siltation. The algorithm achieved accuracy as high as 84.53%, after cross-validation of the results.

Lacking of oxygen (O_2_) has also been a problem for developing countries during the COVID-19 pandemic. As O_2_ is a scarce and expensive resource, an adequate adjustment of oxygen therapy is primordial. Manual titration has showed significant limitations, as healthcare personnel in the hospitals dedicate a significant amount of time to this activity, achieving suboptimal results. Overdosing is common, contributing to poor therapeutic compliance and increasing the financial burden. Currently, the Free O_2_® (Oxynov, Quebec, Canada) is the device with the most scientific evidence on O_2_ titration for spontaneously breathing patients. It takes O_2_ directly from the wall, achieving flows up to 50l/min. The algorithm can be set to the desired peripheral capillary oxygen saturation (SpO_2_) range, and the device will regulate the flow, aiming for the established preset. It has demonstrated potential on sparing O_2_ and achieving the aimed SpO_2_ [9]. Another study addressed autonomous control of inspired oxygen concentration during mechanical ventilation in critically injured trauma patients [10]. The investigators operated an algorithm that was incorporated to the mechanical ventilators, with a target of SpO2 of 94 ± 2%, and prepared to increase inspired fraction of oxygen rapidly if SpO_2_ was < 88% for more than 10 seconds. Oxygen usage was reduced by 44% during autonomous control, and there were no differences in total duration of SpO_2_< 88% when compared to manual titration.

In the next years, the mechanical ventilator will be integrated with all the other bedside technologies within the ICU, and protocols will be part of the basic operation of the machine. The idea is to replicate the intensive care physician´s expertise, and providing the non-specialist with information, and not lists of random data. Decision support will be given, and at some point, decisions will be made: the program will effectively change the ventilators variables, notifying the physician about the problem and possible other solutions. However, implementing artificial intelligence (AI) in low and middle income countries involves building basic infrastructure for technology, as electricity and internet, which is still a major obstacle in the poorest areas of the globe. Given the shortage of investments, there is skepticism about the value of introducing AI in resource-constrained settings. Nonetheless, even though AI can demand a sizable amount of funds at first hand, it can easily pay for itself, by revolutionizing the provision of healthcare services. Human resources are also extremely important, since automated algorithms can turn into dangerous tools in the wrong hands. Doctors will have to integrate AI into their workflow, assimilating primary notions of ML and acquiring technological proficiency.

Respiratory care for the critically ill is a complex and difficult task. The COVID-19 pandemic has brought to light the challenge that healthcare institutions and governments will face in the next decades, since viral pandemics are known for their recurrence. Artificial intelligence constitutes a breath of hope that the world will be better prepared next time, as we will testify its flourishing in the next years. Although it may seem an expensive solution, these technologies could make a big return, either in human lives or money. Sooner than we think, governments and global entities will find themselves in a crossroad: is artificial intelligence too expensive to be considered, or too good to be forgotten in developing countries? Unprecedent occasion for expanding datasets are at the doorstep, as these will be the last nations to enter the new era. Endless opportunities for research are at hand, as medicine is about to be rediscovered.
